# Study Protocol for the Evaluation of Individual Psychological Interventions for Family Caregivers of Advanced Cancer Patients

**DOI:** 10.3389/fpsyg.2020.587627

**Published:** 2021-01-20

**Authors:** Min Yang, Rui Sun, Yanfeng Wang, Haiyan Xu, Baohua Zou, Yanmin Yang, Minghua Cong, Yadi Zheng, Lei Yu, Fei Ma, Tinglin Qiu, Jiang Li

**Affiliations:** ^1^Department of Comprehensive Oncology, National Cancer Center/National Clinical Research Center for Cancer/Cancer Hospital, Chinese Academy of Medical Sciences and Peking Union Medical College, Beijing, China; ^2^Office of Cancer Screening, National Cancer Center/National Clinical Research Center for Cancer/Cancer Hospital, Chinese Academy of Medical Sciences and Peking Union Medical College, Beijing, China; ^3^Department of Internal Medicine, National Cancer Center/National Clinical Research Center for Cancer/Cancer Hospital, Chinese Academy of Medical Sciences and Peking Union Medical College, Beijing, China; ^4^Division of Medical Services, National Cancer Center/National Clinical Research Center for Cancer/Cancer Hospital, Chinese Academy of Medical Sciences and Peking Union Medical College, Beijing, China

**Keywords:** N-of-1 trial, advanced cancer patients, psychological intervention, family caregivers, individual effectiveness

## Abstract

**Background:** Both anxiety and depression in family caregivers (FCs) of advanced cancer patients are common, and they have a negative influence on both the FCs and the patients. Some studies suggested that a variety of interventions could alleviate the psychological symptoms of FCs. However, there is no consensus on much more effective methods for intervention, and relatively high-quality research is blank in psychological problems of these population in China. The validity of mindfulness-based stress reduction (MBSR) and psychological consultation guided by the needs assessment tool (NST) in the psychological status of caregivers will be compared in this study to select a more suitable intervention for the FCs of advanced cancer patients in China.

**Methods and Analysis:** A randomized N-of-1 trial would be conducted at the Cancer Hospital, Chinese Academy of Medical Sciences. Fifty eligible FCs of advanced cancer patients will be recruited, and all will receive three cycles of psychological intervention treatment, with each cycle including both of MBSR and psychological consultation guided by the NST. MBSR and psychological consultation guided by the NST will be compared with each other in each cycle, and the intervention sequence will be based on the random number table generated after the informed consent has been completed. Each treatment period is 2 weeks, and the interval between different treatment cycles or treatment periods is 1 week. The self-reported scales are measured at the beginning and end of each treatment period, including the Self-Rating Anxiety Scale (SAS), the Self-Rating Depression Scale (SDS), Distress Thermometer (DT), Zarit Burden Interview (ZBI), Chinese version of the Medical Outcomes Study 12-item Short Form (C-SF-12), and Family Carer Satisfaction with Palliative Care scale (FAMCARE-2).

**Dissemination:** The protocol of the study was approved by the Institutional Review Board of the Ethical Committee of the Cancer Hospital, Chinese Academic of Medical Science. The results will be published in a peer-reviewed medical journal. The study is registered at Chinese Clinical Trials Registry with the trial registration number chiCTR2000033707. This study employs an innovative methodological approach on the effectiveness of MBSR and psychological consultation guided by the NST for psychological status of FCs of advanced cancer patients. The findings of the study will be helpful to provide high-quality evidence-based medical data for psychological intervention of FCs of advanced cancer patients, and guide clinicians on best quality treatment recommendations.

## Introduction

Cancer has become a major health problem worldwide, with an estimated 18.1 million new cases and 9.6 million deaths in 2018 (Bray et al., 2018). In China, 3.3 million new cases and 2.4 million deaths occurred in 2015 (Zheng et al., 2019). In the past 10 years, the incidence and mortality rate of cancer in China have maintained annual growth rates of 3.9 and 2.5%, respectively (Chen et al., 2018). Much of the rising burden lead to cost and society problem besides medical problems, as well as psychosocial issues. Cancer is a severe stressful event for both patients and family caregivers (FCs), and cancer is the third most common reason for adult caregiving (Kent et al., 2016; Sun et al., 2019). FCs have to offer physical and emotional care for cancer patients, and they suffer financial, social, physical, and mental burdens (Geng et al., 2018; Wang et al., 2018; Alam et al., 2020; Duimering et al., 2020). FCs of advanced cancer patients are at greater risk for psychological problems or distress than FCs of other kinds of patients, which is likely due to the deterioration of the disease, serious adverse reactions related to anticancer treatment, and more intolerable severe symptoms at the end of life (Tang, 2006; Williams and Mccorkle, 2011; Oechsle et al., 2019; Teixeira et al., 2019; Lin et al., 2020). Psychological problems, mainly anxiety and depression, also have profound adverse effects on the quality of life QOL of both patients and caregivers (Sun et al., 2019; Teixeira et al., 2019). The prevalence rates of significant anxiety and depression among FCs of advanced cancer patients vary largely, from 40 to 47% and from 16 to 67%, respectively (Oechsle et al., 2019; Teo et al., 2019; Alam et al., 2020).

Family-centered palliative care may improve the outcomes of both FCs and patients (Kent et al., 2016; Ferrell et al., 2017). However, both research and practice evidence are mostly limited to patients, and the psychological needs and care of FCs are often neglected (Kent et al., 2016). Few studies on psychological interventions for FCs in advanced cancer patients are found in the literature, but the number is growing. Some studies documented improvements in anxiety and depression resulting from early and standard psychological interventions (Fu et al., 2017; Alam et al., 2020). Few high-level evidence-based medical studies on the effectiveness of different interventions in China have been reported, and there is no consensus or guideline on psychological intervention among FCs of advanced cancer patients (Fu et al., 2017; Geng et al., 2018).

At present, mindfulness-based stress reduction (MBSR) and psychological consultation guided by the needs assessment tool (NST) are relatively widely used among FCs of advanced cancer patients (Zhang et al., 2015; Zimmermann et al., 2018). Previous studies found that both classic 8-week MBSR sessions and abbreviated 2-week sessions were effective in reducing depression, anxiety, and distress (Grossman et al., 2004; Khoury et al., 2015; Quinones and Griffiths, 2019; Wathugala et al., 2019). MBSR helps FCs focus on the current physical and mental state, primarily through meditation, to accept the state in a positive way. The psychological consultation guided by the NST is carried out by professionals after they assess the unmet needs of FCs through scales. MBSR is a kind of group intervention, which does not aim at individual specific problems. Psychological consultation is a one-to-one form, which requires more manpower and time. So far, there are no clinical trials directly comparing these two interventional techniques among FCs. Most previous studies are conventional prospective randomized controlled studies comparing the differences between the groups, but personalized intervention and assessment are the keys to explain the effectiveness of psychological intervention.

N-of-1 trials, which are within-patient randomized multi-period crossover trials that compare two different therapeutic strategies, provide the highest level evidence of intervention effects for individuals, and enhance precision when intervention effects are heterogeneous between individuals (Mirza and Guyatt, 2018; Porcino et al., 2020). At least 2 cycles with 4 periods are needed to compare the effectiveness and give much more accurate. In our study, because of the high quality of compliance, 3 cycles with 6 periods are designed and are available.

Therefore, we plan to conduct this study through an N-of-1 trial, in which the bias resulting from the difference between individuals can be minimized to the greatest extent. In the protocol, the design of this N-of-1 trials is given in detail. The main purpose of the study is to compare the effect of MBSR and psychological consultation guided by the NST on the psychological well-being and QOL of FCs of advanced cancer patients.

## Methods

### Study Design

**Figure 1:**
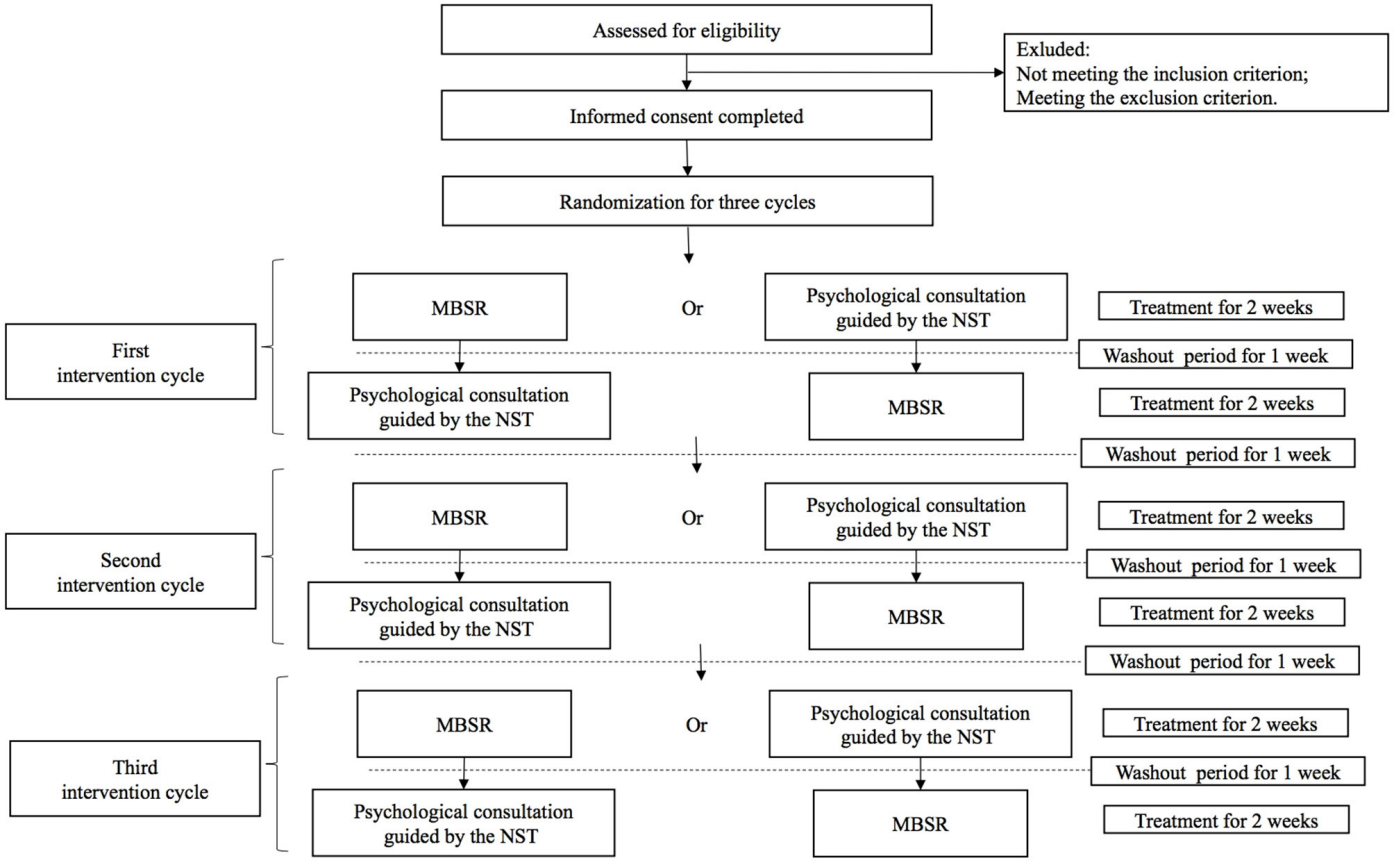
The flowchart of the study process.

This study is a single-case randomized controlled trial that will be conducted at the Cancer Hospital, Chinese Academy of Medical Sciences. Each FC of advanced cancer patients will receive three cycles of psychological intervention treatment, with each cycle including both of MBSR and psychological consultation guided by the NST. MBSR and psychological consultation guided by the NST will be compared with each other in each cycle, and the intervention sequence will be based on the random number table generated after the informed consent has been completed. Each treatment period is 2 weeks, and the interval between different treatment cycles or treatment periods is 1 week. The outcomes will be measured at the beginning and end of each treatment period (see Figure 1).

### Study Participants

Clinicians trained in palliative care, psychologists, and nurses will be recruited from the comprehensive oncology department of Cancer Hospital, Chinese Academy of Medical Sciences. Four clinicians and five nurses should be included. Three-round with 6 h training based on panel discussion will be given by the principle investigators (JL and MY). All the training including the process and interventions which are based on correct translation and panel discussion would be given by Chinese.

Fifty eligible FCs of advanced cancer patients will be recruited consecutively by advertising in the Cancer Hospital, Chinese Academy of Medical Sciences. During a 12-month period, FCs of advanced cancer patients will be consecutively assessed for study eligibility within 72 h after the patient's first admission to the comprehensive oncology ward. Once the FC completes the assessment for eligibility, the principle investigator of the research and the authorized clinical research coordinator will execute the strict screening and check the following standard inclusion criteria and exclusion criteria personally. For a period of 1 consecutive week, participants will be randomly assigned for three treatment cycles including the sequences of six intervention periods.

Inclusion criteria:

- Age between 18 and 75 years old;
- the patient has been diagnosed with advanced cancer;
- the expected survival time of the cancer patient is >6 months, according to the subjective assessment of two palliative care specialists;
- FC, taking the main responsibility for care, is aware of the patient's condition as a family member of the patient;
- for the FC, either the Self-Rating Anxiety Scale (SAS) score or the Self-Rating Depression Scale (SDS) score is >50 (Zung, 1965, 1971);
- FC has no communication or language barriers and can complete the questionnaire or scale independently;
- FC signs the informed consent form voluntarily.

Exclusion criteria:

- FC has a history of suicide or mental illness;
- FC is taking anti-anxiety medications or antidepressants;
- FC has potential risk factors such as mental, psychological, family, social or geographical factors that hinder the research programme;
- FC is a pregnant or lactating woman;
- FC is suspected or diagnosed by psychiatrists as mental disorders such as schizophrenia;
- any other reasons that the FC cannot complete the study in the judgement of the investigator.

### Sample Size Calculation

The sample size was calculated based on the previous research of Senn, and according to the power of 80% (Senn, 2002, 2019). The assumption of a minimally important difference of 0.4 SDs on the SAS and SDS interference scale (Liu and Yang, 2019). Assuming a 5% dropout rate, using a 2-group *t*-test with 2-sided alpha equalling 0.05, 300 periods (50 participants and 6 periods for each person) would achieve study objectives.

### Participants Recruitment

In-patient broadcasting, flyers and science popularization presentations will be used to raise the public awareness of the study. Once the FC shows some interest of the study, informed consent and detailed explanation to recruit into the study will be given.

### Interventions

MBSR was developed by Kabat-Zinn at the University of Massachusetts Medical Center in 1979, and it has a standardized operating procedure (Zimmermann et al., 2018). In MBSR practice, individuals are encouraged to pay attention to what is happening in the moment, in a non-judgemental way, without relying on previously formed schemas. The objective of this approach is to help individuals change the way they think through mindfulness meditation and exercises. The abbreviated 2-week MBSR mainly includes Body Scan Mediation and Sitting Mediation used audio recordings from an online version of the Kabat-Zinn course (made by a certified MBSR instructor) and previous studies (Quinones and Griffiths, 2019; Wathugala et al., 2019; Potter, 2020). On the first day (the start of week 1), the participant will be taught what MBSR and Body Scan Mediation are by an expert, and all participants will practice daily while being individually guided by the special audio during days 2–14. For days 2–7, they will practice a 5-min Body Scan Mediation each day. On day 8 (the start of week 2), the participant will learn what Sitting Mediation is, and they will practice a daily 5-min Sitting Meditation during days 9–14. Meanwhile, each participant will receive an audio document and an operating manual that summarizes key points of the six sessions and clarifies homework requirements. The participants should complete the practice according the guidance of the daily audio document and record on the manual faithfully.

The psychological consultation refers to the one-to-one consultation after the analysis according to the Chinese version of the Comprehensive Needs Assessment Tool in cancer for Caregivers (CNAT-C) (Zhang et al., 2015). The CNAT-C was developed in 2011, including seven dimensions and a total of 41 items, and it has been verified to have good reliability and validity among caregivers of cancer patients in China. It was reported by Zhang that the total Cronbach's α coefficient was 0.94 and the dimensional Cronbach's α coefficient was 0.61–0.93. Consultation focusing on problems found or raised through the CNAT-C, is generally once a week, approximately 50 min each session. The specific frequency and time arrangement depend on the specific situation of the participant, and every counseling session will be recorded.

Both interventions have manuals and will be delivered by clinicians trained in the intervention for which they are responsible. All the trainers have corresponding qualification certificate and operation experience. The principle investigator of the research/ authorized clinical research coordinator will review the record of each intervention, and give back to the trainers.

### Randomization and Allocation

Random grouping will be performed by a third party who is not directly involved in the trial according to the random number table method. Computer-generated random numbers will be made and secured by the Information Technology team with SAS 9.4 software. The random number will be kept in an envelope.

### Blinding

The assessment staff and data analysts will be blind to the participants' sequence allocations. The allocation sequence would be concealed until interventions are assigned.

### Patient Safety and Quality Control

The participants will be free to change their mind about participation at any time and will be advised to see their individual doctors to discuss future routine care. The researchers can withdraw a participant at any time if serious psychological problems occur or if the participant presents with any reason to stop the psychological intervention. Termination criteria are as follows: (1) the participant complete the intervention and follow-up; (2) the end date of the study is December 31, 2021; (3) terminate the trial at the request of the ethics committee or national government department. Adherence to trial treatment will be assessed by (1) self-reporting scales by FCs of advanced cancer patients, including SAS, SDS, DT, and so on, as part of outcome data collection and (2) the record of the psychological interventions.

### Outcomes

#### Primary Outcome

The primary outcome is the psychological status, assessed by the degree of anxiety and depression measured at the beginning and end of each treatment period. The degree of anxiety and depression will be measured by the SAS and SDS, and both of them are self-report questionnaires with 20 items rated on a four-point scale (Zung, 1965, 1971).

#### Secondary Outcomes

The secondary outcomes are the distress, caregiver burden, quality of life, and satisfaction with care, assessed by the corresponding scales measured at the beginning and end of each treatment period. Distress will be measured using the Distress thermometer (DT), which is a self-reporting instrument with two parts: a visual image scale ranging from 0 (no distress) to 10 (extreme distress) and a problem list with 36 questions (Riba et al., 2019). Caregiver burden will be measured using the Zarit Burden Interview (ZBI), which is a 22-item self-reporting questionnaire, rated on a five-point Likert scale, from 0 (“never”) to 4 (“nearly always”) (Zarit et al., 1980). QOL will be assessed with the Chinese version of the Medical Outcomes Study 12-item Short Form (C-SF-12), which is a 12-item scale containing eight subscales and two dimensions (Ware et al., 1996). Satisfaction with care will be measured using the Family Carer Satisfaction with Palliative Care scale (FAMCARE-2), which is a 20-item scale developed to measure family members' satisfaction with the delivery of palliative care (Kristjanson, 1993).

### Data Collection

Baseline data will include demographic information, contact details, and psychological assessment. The intervention outcomes including multiple scales, will be measured at the beginning and end of each treatment period. These data will be collected in paper form or WeChat form, which is a popular and convenient social networking application for mobile phones in China. Each participant will be interviewed, followed up and assessed by the specially appointed training nurses using print scales or electronic scales. The electronic scales through WeChat app will be sent to the participants by the training nurses. For the WeChat app, application by ID card of principle investigators for the official account has been submitted, and after obtaining the qualification authorization, the information which had been filled by participants can be checked and downloaded through the account. A database management based on Access 3.0 would be made. Two trained nurses will entry the data when data available. Each data collection takes about 20 min for participants. These data will be entered directly into the electronic trial database.

### Participant Timeline

The study is expected to end on December 31, 2021, and the expected recruitment period is November 2020 to June 2021. Each participant will spend about 5 months from start of his/her individual assessment until the end of the individual treatment, 12 weeks of which are 6 treatment periods and 5 weeks are 5 intervals between different treatment cycles or treatment periods.

### Statistical Methods

For each cycle, each washout periods, and each person, the data from the baseline will be managed to conduct analysis. Outcomes will be analyzed with longitudinal mixed effects models combining baseline and each period's measurements using time, treatment, and a time-by-treatment interaction as fixed effects, and clinician and patient as random effects. A Bayesian multi-level random effects model on the outcome will be used to combine in incorporating serial correlation and other covariates for each participant by WinBUGS software (Spiegelhalter et al., 2003).

Traditional statistical method also will be used. The interactions between the intervention and covariates including age, sex, marital status, education, employment status, etc. will conduct exploratory analyses. The paired *t*-test will be used to compare the continuous quantitative data between the two groups, and the paired chi-squared test will be used to classify the data. The measurement data will be expressed as X ± s. A *P* < *0.05* will be considered statistically significant.

### Data Monitoring

An independent data monitoring team has been appointed for this trial to oversee safety and validity monitoring. The data monitoring team will review accumulating data on a regular basis from the ongoing trial, and they will review the validity and scientific merit of the trial. An independent statistician is appointed to provide the analysis service required by the data monitoring team. All trial-related and source documents must be kept for 5 years after the end of the trial.

### Harms

Although the study is an intervention study, it will not cause any expected risk or harm to the subjects or increase the medical expenses for the subjects. In the previous studies, there were no significant relative harms of the two intervention methods for psychological status (Botha et al., 2015; Fu et al., 2017; Hirshberg et al., 2020). Through the research, the psychological status of subjects may be dynamically evaluated and improved by intervention.

## Ethics Statement

The research protocol involving human participants were reviewed and approved by the Institutional Review Board at Ethical Committee of Cancer Hospital, Chinese Academic of Medical Science. Written informed consent will be obtained from participants to participate in the study.

## Informed Consent

Informed consent will be provided and obtained by the research nurses.

## Confidentiality

Participant data will be accessed only by authorized researchers. Every research member has a duty of confidentiality, and no relative data including demographic information, contact details, psychological assessment and so on, will be disclosed outside the research site.

## Dissemination Plans

The trial results will be published in peer-reviewed journals. All publications will follow the Consolidated Standards of Reporting Trials statement. Links to the publication will be provided in all applicable trial registers. The dissemination of results to patients will take place through the trial website (http://www.chictr.org.cn/index.aspx), journals and related media. Authorship for all publications will be based on the criteria defined by the International Committee of Medical Journal Editors.

## Author Contributions

MY and JL led the study design and will coordinate the overall work of the study. MY, FM, JL, and TQ drafted the study protocol and will supervise clinical training work at hospital. JL, YZ, and TQ will contribute to the data analysis and management. YW, RS, HX, BZ, MC, and LY contributed to quality control in intervention technology. All authors reviewed and revised the manuscript, and approved the final version for publication.

## Conflict of Interest

The authors declare that the research was conducted in the absence of any commercial or financial relationships that could be construed as a potential conflict of interest.
